# Associations between cigarette smoking and biliary tract cancer by anatomic subsite and sex: a prospective cohort study in Japan

**DOI:** 10.1007/s10552-022-01600-y

**Published:** 2022-08-27

**Authors:** Yingsong Lin, Sayo Kawai, Tae Sasakabe, Michiko Kurosawa, Akiko Tamakoshi, Shogo Kikuchi, The JACC Study Group

**Affiliations:** 1 Department of Public Health, Aichi Medical University School of Medicine, Aichi, 480-1195 Japan; 2 Department of Epidemiology and Environmental Health, Juntendo University Faculty of Medicine, Tokyo, Japan; 3 Department of Public Health, Hokkaido University Graduate School of Medicine, Sapporo, Japan

**Keywords:** Biliary tract cancer, Cigarette smoking, Cohort study, Risk

## Abstract

**Purpose:**

Biliary tract cancer (BTC) has not been considered a tobacco-related cancer, largely because of inconclusive results from epidemiological studies. We herein evaluate the association between cigarette smoking and risk of death from BTC by anatomic subsite and sex using data from a large, prospective cohort study in Japan.

**Methods:**

The present study included 97,030 Japanese individuals who were enrolled in 1988–1990 and followed until 31 December 2009. Cox proportional hazards regression models were used to estimate relative risks (RRs) and 95% confidence intervals (CIs) for the association of BTC with cigarette smoking, including smoking status, number of cigarettes smoked per day, and pack-years of smoking.

**Results:**

During a mean follow-up of 16.2 years, we documented 484 deaths (187 from gallbladder cancers and 297 from cancers of other and unspecified biliary tract parts). After adjustment for sex, age, body mass index, alcohol consumption, and history of gallstones, current smokers had a higher risk of death due to BTC (RR = 1.35, 95% CI = 1.01–1.79) than never smokers. In the analyses by anatomic subsite, current smoking was associated with an increased risk of death from gallbladder cancer (RR = 1.89 95% CI = 1.19–3.02), whereas no evidence of an association was noted for cancers of other and unspecified biliary tract parts (RR = 1.10, 95% CI = 0.77–1.58). Moreover, mortality risk increased with an increasing number of cigarettes smoked per day and pack-years of smoking, particularly for gallbladder cancer in men.

**Conclusion:**

Cigarette smoking is associated with an increased risk of death from BTC, particularly gallbladder cancer, in Japanese men.

## Introduction

Biliary tract cancer (BTC) comprises a spectrum of invasive adenocarcinomas that arise from the bile duct epithelium [1]. BTC is subclassified into cancers of the gallbladder, intrahepatic bile duct, and extrahepatic bile duct, depending on the location of the tumor [2]. Overall, BTC is uncommon in high-income countries, but incidence rates vary widely according to sex, geographical region, and anatomic subsite [3]. The incidence/mortality burden of BTC is relatively higher in Japan, where it is the 6th leading cause of cancer-related death, accounting for approximately 18,000 deaths annually [4].

Unlike other gastrointestinal tract cancers, such as pancreatic and colorectal cancers, BTC has been understudied, and its etiology remains largely unknown. Furthermore, BTC is often diagnosed at an advanced stage, with few viable treatment options, resulting in a poor prognosis (5-year survival rate < 30%) [5]. Identifying modifiable risk factors is thus critical for informing prevention and early detection of this malignancy. Although previous studies have linked cigarette smoking and alcohol consumption with BTC risk, findings have been contradictory or inconclusive [6, 7]. One major reason is that most of the studies were limited by a small number of BTC cases, making it difficult to obtain robust risk estimates and to analyze subtype-specific associations. Nevertheless, collaborative efforts to overcome this limitation are providing results. In 2019, a pooled analysis of 26 prospective studies revealed that smoking is associated with increased risks of all BTC subsites, except for gallbladder cancer, and that alcohol use increases the risk of intrahepatic bile duct cancer [8]. These findings suggest that the association of BTC with cigarette smoking or alcohol consumption differs by anatomic subsite. In parallel, genetic studies have provided strong evidence that BTC located at different anatomic sites exhibits distinctive profiles of driver mutations [9, 10]. Together, there is an increasing appreciation of the heterogeneity of BTC based on anatomic subsite with respect to risk factors, genetic alterations, and clinical presentation [3]. It is therefore desirable to explore the etiology of BTC by tumor location in the biliary tract.

In 2008, we reported a positive association between smoking and the mortality risk of gallbladder cancer based on follow-up data from 1988 to 2003 of the Japan Collaborative Cohort Study (JACC study), a large prospective cohort study involving 110,585 middle-aged and elderly Japanese individuals [11]. However, we did not report associations with extrahepatic bile duct cancers or other BTC subtypes. With an extended follow-up period through 31 December 2009, and an updated number of deaths in the same cohort, we re-examined this association according to anatomic subsite and sex.

## Methods

We analyzed data from the JACC Study, the details of which have been described elsewhere [12]. Briefly, 46,395 men and 64,190 women (110,585 in total) aged 40 to 79 years were recruited in 45 areas throughout Japan between 1988 and 1990. A self-administered questionnaire was used to collect information such as demographic characteristics, medical history, anthropometric measurements, and lifestyle factors, among others. The participants were then followed for mortality until 31 December 2009. Because of logistical problems, we discontinued follow-ups prior to 31 December 2009 in 10 areas. Subjects were excluded if they reported a history of cancer or surgical removal of the gallbladder. Those who did not provide valid responses to the questions related to smoking status were also excluded. After exclusions, 97,030 subjects were eligible for the current analysis, with a mean age of 57.3 years at baseline.

Completion of the questionnaire with a signature on the cover page was considered implied consent. However, it was obtained at the group level in a few areas because the concept of informed consent was not popularized during the 1980s in Japan. In that case, the municipality head gave the consent to participation representing the participants living in that area. This study was performed in line with the principles of the Declaration of Helsinki, and was approved by the ethics committees of Hokkaido University and Aichi Medical University School of Medicine.

### Outcome ascertainment

The vital status of the cohort participants was determined through resident registries. The cause of death recorded on death certificates was classified according to the International Classification of Diseases (ICD), 10th Revision. The outcome of interest was death from BTC during the follow-up period, as coded C23 for malignant neoplasm of the gallbladder and C24 for malignant neoplasm of other and unspecified parts of the biliary tract. Information regarding subclassification of C24 was not available for the present analysis, but on the basis of Osaka Cancer Registry data, cases coded as C24.0 (malignant neoplasm of the extrahepatic bile duct) accounted for approximately 80% of the cases coded as C24 [13]. We did not include intrahepatic bile duct cancer (C22.1) because it was coded under liver cancer (C22). During the study period, 5.8% of the participants moved out of the study area.

### Collection of exposure data

At baseline, all study participants were asked whether they were currently smoking cigarettes and whether they had ever smoked in the past. All current and former smokers were asked at what age they started smoking regularly, the number of cigarettes smoked per day, and smoking duration in years. Pack-years of smoking were estimated using data on the number of cigarettes smoked per day and years of smoking.

Information on demographic characteristics and other lifestyle factors (including alcohol use, coffee consumption, and physical activity) was collected through the baseline questionnaire.

### Statistical analysis

We computed person-years of follow-up for each participant from the time of initial enrollment to (a) death from BTC or any other cause, (b) the time of moving out of the study area, or (c) the end of follow-up, whichever occurred first. Individuals who died from causes other than BTC or who moved out of the study area were censored. Cox proportional hazards regression models were used to estimate relative risks (RRs) and 95% confidence intervals (CIs) for the association of BTC with cigarette smoking, including smoking status, intensity (number of cigarettes smoked per day), and cumulative amount (pack-years of smoking). The proportional hazards assumption was checked by adding time-dependent covariates in the Cox model and the results indicated no violation of proportional hazards assumption. We calculated both age- and sex-adjusted RRs and multivariable-adjusted RRs. In multivariable analyses, risk estimates were adjusted for age (continuous), body mass index (less than 20.0, 20.0–22.4, 22.5–24.9, and 25.0 or more), history of gallstones (yes or no), and alcohol consumption (never, former, or current). The percentage of missing values was 4.9% for BMI, 10.7% for history of gallstones, and 3.1% for alcohol consumption. These missing values for each covariate were treated as an additional category.

*p* values for the linear trend test across exposure categories were calculated by assigning a score to each category. The effect modification by sex for the associations between cigarette smoking and the risk of death from gallbladder cancer was evaluated by adding an interaction term in the model. All P values were based on 2-sided tests, and *p* < 0.05 was considered statistically significant. All analyses were conducted using SAS 9.4 (SAS Institute, Cary, NC, USA).

## Results

We documented 484 deaths due to BTC, including 187 from gallbladder cancer and 297 deaths from cancers of other parts (or unspecified) of the biliary tract, during a total of 1,574,855 person-years of follow-up. The characteristics and lifestyle factors of the cohort participants are shown in Table 1. Ever smokers (current smokers and former smokers) accounted for 80% of men and 7% of women. For men, diabetes history and current drinker proportions tended to be higher among ever smokers than never smokers. For women, ever smokers had higher proportions of diabetes history and gallstones.

**Table 1:** Selected characteristics of the JACC Study participants at baseline by smoking status

	Men (*n* = 43,262)				Women (*n* = 53,768)	
Characteristics	Current smokers (*n* = 23,090)	Former smokers (*n* = 11,274)	Never smokers (*n* = 8898)	Current smokers (*n* = 2943)	Former smokers (*n* = 900)	Never smokers (*n* = 49,925)
Age at cohort entry (mean ± SD)	56.2 ± 10.0	59.8 ± 10.1	57.0 ± 10.3	56.3 ± 10.7	60.3 ± 10.7	57.2 ± 10.0
BMI of 25 or more (%)	15.4	19.9	21.0	21.8	26.4	21.5
History of gallstones (%)	2.6	3.6	3.0	5.6	5.7	3.5
History of diabetes (%)	5.7	7.5	5.3	4.3	6.1	3.4
Current drinkers (%)	75.6	71.5	67.6	41.2	41.1	21.4
Walking for 1 h or more (%)	48.1	44.3	47.3	46.0	40.1	48.4
College/university education or more (%)	39.7	41.7	40.3	31.1	31.9	36.2

Table 2 presents associations of risk of mortality from BTC with smoking status, intensity, and cumulative exposure based on anatomic site and sex. When BTC was analyzed as a group, current smokers had an increased risk of death from BTC (multivariable-adjusted RR = 1.35, 95% CI = 1.01–1.79) when compared with never smokers after adjustment for covariates. The results remain unchanged when we removed history of gallstones from covariates in the model. In analyses by anatomic site, current smoking was associated with an increased risk of death from gallbladder cancer (multivariable-adjusted RR = 1.89 95% CI = 1.19–3.02), but there was no evidence of an association for cancers of other and unspecified parts of the biliary tract (multivariable-adjusted RR = 1.10, 95% CI = 0.77–1.58). Regarding gallbladder cancer, risk of death increased significantly with high-intensity smoking, with a 2.3-fold higher mortality risk among individuals smoking 20 or more cigarettes per day. Similarly, increased cumulative exposure to cigarette smoke was associated with a higher mortality risk, with a RR of 2.15 (95% CI 1.21–3.83) for individuals who smoked 1 pack of cigarettes for 40 years or more. These observed associations did not change materially when the model was additionally adjusted for history of diabetes or education.

**Table 2:** Associations of biliary tract cancer with cigarette smoking by anatomic subsite and sex

	Biliary tract cancer (excluding intrahepatic bile duct)			Gallbladder			Cancers of other and unspecified parts of biliary tract		
	Deaths	Age-adjusted RR	Multivariate-adjusted RR	Deaths	Age-adjusted RR	Multivariate-adjusted RR	Deaths	Age-adjusted RR	Multivariate-adjusted RR
Overall									
Never	253	1.00	1.00	97	1.00	1.00	156	1.00	1.00
Former	82	1.21 (0.88–1.66)	1.15 (0.83–1.60)	28	1.44 (0.84–2.48)	1.34 (0.76–2.36)	54	1.08 (0.73–1.60)	1.05 (0.70–1.57)
Current	149	1.33 (1.03–1.79)	1.35 (1.01–1.79)	62	1.94 (1.24–2.05)	1.89 (1.19–3.02)	87	1.09 (0.79–1.55)	1.10 (0.77–1.58)
Cigarettes/day									
0–19	53	1.14 (0.81–1.60)	1.13 (0.80–1.60)	21	1.54 (0.88–2.68)	1.43 (0.80–2.55)	32	0.98 (0.63–1.53)	1.02 (0.66–1.60)
≧20	94	1.51 (1.10–2.07)	1.49 (1.08–2.05)	39	2.28 (1.35–3.78)	2.27 (1.32–3.89)	55	1.23 (0.83–1.84)	1.22 (0.81–1.84)
Trend P		0.009	0.01		0.003	0.004		0.23	0.27
Pack-years									
Never smokers	253	1.00	1.00	97	1.00	1.00	156	1.00	1.00
1–19	45	1.28 (0.89–1.83)	1.23 (0.85–1.77)	14	1.31 (0.70–2.44)	1.16 (0.60–2.25)	31	1.20 (0.78–1.85)	1.19 (0.77–1.85)
20–39	79	1.21 (0.87–1.68)	1.19 (0.85–1.67)	29	1.58 (0.90–2.78)	1.55 (0.86–2.78)	50	1.05 (0.71–1.57)	1.04 (0.70–1.57)
≧40	84	1.42 (1.02–1.98)	1.37 (0.97–1.92)	37	2.25 (1.30–3.92)	2.15 (1.21–3.83)	47	1.10 (0.73–1.65)	1.07 (0.70–1.62)
Trend P		0.09	0.09		0.004	0.008		0.78	0.88
Men									
Never	44	1.00	1.00	12	1.00	1.00	33	1.00	1.00
Former	79	1.28 (0.89–1.86)	1.22 (0.83–1.78)	26	1.52 (0.77–3.01)	1.34 (0.66–2.71)	53	1.20 (0.77–1.86)	1.18 (0.75–1.85)
Current									
1–19	45	1.21 (0.80–1.84)	1.18 (0.77–1.80)	15	1.47 (0.69–3.14)	1.26 (0.57–2.78)	30	1.12 (0.68–1.85)	1.15 (0.69–1.89)
≧20	89	1.58 (1.10–2.28)	1.54 (1.06–2.24)	38	2.65 (1.39–5.09)	2.58 (1.33–5.01)	51	1.21 (0.77–1.89)	1.17 (0.74–1.85)
Trend P		0.01	0.02		0.003	0.003		0.44	0.54
Pack-years									
Never smokers	44	1.00	1.00	12	1.00	1.00	32	1.00	1.00
1–19	36	1.37 (0.88–2.12)	1.30 (0.82–2.04)	9	1.29 (0.54–3.07)	1.03 (0.40–2.62)	27	1.39 (0.83–2.32)	1.38 (0.82–2.32)
20–39	76	1.26 (0.87–1.84)	1.22 (0.84–1.79)	27	1.71 (0.86–3.38)	1.62 (0.81–3.25)	49	1.10 (0.71–1.72)	1.08 (0.68–1.70)
≧40	82	1.48 (1.03–2.14)	1.40 (0.97–2.04)	37	2.42 (1.26–4.64)	2.24 (1.16–4.35)	45	1.13 (0.72–1.78)	1.09 (0.68–1.73)
Trend P		0.06	0.10		0.004	0.007		0.81	0.98
Women									
Never	209	1.00	1.00	85	1.00	1.00	124	1.00	1.00
Former	3	0.73 (0.23–2.28)	0.74 (0.24–2.34)	2	1.23 (0.30–5.00)	1.26 (0.31–5.21)	1	0.40 (0.06–2.87)	0.41 (0.06–2.93)
Current									
1–19	8	1.08 (0.53–2.19)	1.08 (0.53–2.34)	6	1.97 (0.86–4.51)	1.96 (0.86–4.51)	2	0.46 (0.11–1.86)	0.46 (0.11–1.88)
≧20	5	2.02 (0.83–4.90)	1.75 (0.79–4.79)	1	0.93 (0.13–6.72)	0.92 (0.13–6.66)	4	2.85 (1.05–7.72)	2.75 (0.9997–7.58)
Trend P		0.19	0.23		0.33	0.38		0.36	0.39
Pack-years									
Never smokers	209	1.00	1.00	85	1.00	1.00	124	1.00	1.00
1–19	9	1.12 (0.59–2.18)	1.10 (0.56–2.16)	5	1.50 (0.61–3.69)	1.48 (0.59–3.70)	4	0.85(0.31–2.30)	0.83 (0.30–2.28)
20–39	3	0.94 (0.30–2.94)	0.94 (0.30–2.96)	2	1.12 (0.28–4.56)	1.13 (0.28–4.68)	1	0.53 (0.08–3.82)	0.53 (0.07–3.83)
≧40	2	1.65 (0.41–6.67)	1.59 (0.39–6.44)	-	-	-	2	2.73 (0.69–11.10)	2.53 (0.62–10.40)
Trend P		0.61	0.66		0.54	0.55		0.75	0.79

Adjusted for sex, age, history of gallstones, body mass index, and alcohol drinking

*RR* Relative risk; *CI* Confidence interval

In the analyses stratified by sex, both high-intensity smoking and pack-years of smoking were associated with increased risks of death from gallbladder cancer in men. In contrast, no apparent increase in mortality risk was noted for female current smokers. We did not observe any evidence of effect modification by sex for the associations between cigarette smoking and the risk of death from BTC (*p* = 0.62).

Although increased cumulative exposure to cigarette smoke (40 pack-years or more) was associated with an increased risk of malignant neoplasm of other and unspecified parts of the biliary tract, the associations observed were statistically nonsignificant.

## Discussion

In this large Japanese cohort study with an extended follow-up period and updated number of deaths, current smokers were found to have a 35% higher risk of death from BTC than never smokers. Furthermore, the risk among current male smokers increased with an increasing number of cigarettes smoked per day and cumulative smoking.

In general, the magnitude of associations between smoking and BTC remains uncertain. The majority of previous studies addressing smoking-BTC associations have been small case–control studies, which were limited to the detection of modest associations and also prone to selection bias and recall bias [14]. Three cohort studies [7, 11, 15] in Japan, including our previous report [11], have examined smoking-BTC associations, but the findings were inconclusive. We herein corroborate our previous results and provide evidence of a dose–response relationship between smoking intensity and cumulative exposure and risk of death from BTC in men. Furthermore, in the analyses by anatomic subsite, smoking was significantly associated with risk of death from gallbladder cancer in men, with a 2.4-fold excess mortality risk for high-intensity current smokers (20 or more cigarettes smoked per day). In contrast, there was no evidence of an association between smoking and cancers of other and unspecified parts of the biliary tract, the majority of which were presumed to be extrahepatic bile duct cancers. These findings suggest etiologic heterogeneity across BTC subtypes, highlighting a role for cumulative exposure to cigarette smoke in the pathogenesis of gallbladder cancer. Nonetheless, our results contrast with those of a pooled analysis of 26 cohort studies, in which smoking was associated with an increased risk of all BTC subtypes, with the exception of gallbladder cancer [8]. It is worth noting that most cohorts included populations of European ancestry, and therefore, it remains unclear whether this result can be applicable to Asian populations. We also noted that 3 of 4 cohort studies from East Asia included in that pooled analysis showed an increased risk among current smokers, though the associations were not statistically significant.

Although pancreaticobiliary maljunction and gallstone disease have been implicated as strong risk factors, risk factors for extrahepatic bile duct cancers are poorly understood [16]. We did not find any significant associations between cigarette smoking and risk of death due to cancers of other (or unspecified) parts of the biliary tract, including mainly extrahepatic bile duct cancers. Regardless, smoking was shown to be associated with 23% elevated risk in a previous meta-analysis study based on 11 case–control studies and 1 cohort study [14]. As there is a paucity of prospective cohort study data as well as a lack of evidence on smoking intensity and cumulative exposure, the causal relationship remains unclear, and further investigation is warranted.

Although BTC has not been established as being caused by cigarette smoking, growing evidence suggests that current smokers are at increased risk of BTC, with associations for each subtype remaining controversial [17]. Plausible mechanisms have been proposed to explain the increased risk associated with smoking. Regarding tobacco-related cancers, such as lung cancer and esophageal cancer, a mechanism centered on DNA adduct-induced mutations in driver genes is well accepted [18]. Repeated exposure to tobacco carcinogens, such as nitrosamine and polycyclic aromatic hydrocarbons, results in the formation of different types of DNA adducts in target tissues, among which some unrepaired DNA adducts may lead to mutations in critical genes that drive carcinogenesis. This mechanism of action appears to apply to BTC, as previous work has discovered the presence of higher levels of DNA adducts in intrahepatic bile duct tumor tissues than in non-cancer control tissues [19]. The direct or indirect toxic effects of tobacco carcinogens on the bile duct likely play a role in the pathogenesis of BTC. An alternative mechanism is that smoking and other factors, such as *Helicobacte*r species infection, may be associated with chronic inflammation in the biliary system, contributing to BTC risk [20].

One strength of our study is its large size, which enabled us to examine the risk of death from BTC associated with different measures of smoking based on anatomic subsite and sex. Furthermore, we were able to adjust for important confounding factors such as gallstones, thereby providing more robust risk estimates for smoking-BTC associations.

Our study also has several limitations. First, the possibility that the observed associations with BTC were influenced by unknown confounding factors cannot be ruled out, given the modest associations when compared with those with other tobacco-related cancers (e.g., lung cancer). Second, updated information on smoking was not collected during follow-up, possibly leading to some misclassification in tobacco use. Third, outcome misclassification might bias the results because deaths caused by BTC were confirmed based on death certificates alone, without further pathologic confirmation, and because an independent group for extrahepatic bile duct cancer could not be analyzed. Finally, the current cohort was characterized by a low proportion of current smokers among women. In particular, the risk estimates for high-intensity smoking were unstable, with wide confidence levels, owing to the few high-intensity smokers. The lack of significant associations for women might be due to inadequate statistical power and should be interpreted with caution.

In conclusion, our study adds to the evidence that high-intensity and cumulative smoking may be associated with an increased risk of death from BTC, particularly gallbladder cancer, in men. BTC located at different anatomic subsites may have distinct etiologies and risk factors. More research is needed to better understand risk factor associations across all BTC subtypes.

## Data Availability

The datasets generated during and/or analyzed during the current study are not publicly available due to the policy of the JACC Study group but are available from the corresponding author on reasonable request.

## References

[1] Valle JW, Lamarca A, Goyal L, Barriuso J, Zhu AX (2017). New horizons for precision medicine in biliary tract cancers. Cancer Discov.

[2] Wardell CP, Fujita M, Yamada T (2018). Genomic characterization of biliary tract cancers identifies driver genes and predisposing mutations. J Hepatol.

[3] Valle JW, Kelley RK, Nervi B, Oh DY, Zhu AX (2021). Biliary tract cancer. Lancet.

[4] Cancer Statistics. Cancer Information Service, National Cancer Center, Japan (Vital Statistics of Japan, Ministry of Health, Labour and Welfare). https://ganjoho.jp/reg_stat/statistics/data/dl/index.html

[5] Ishihara S, Horiguchi A, Miyakawa S, Endo I, Miyazaki M, Takada T (2016). Biliary tract cancer registry in Japan from 2008 to 2013. J Hepatobiliary Pancreat Sci.

[6] Clements O, Eliahoo J, Kim JU, Taylor-Robinson SD, Khan SA (2020). Risk factors for intrahepatic and extrahepatic cholangiocarcinoma: a systematic review and meta-analysis. J Hepatol.

[7] Makiuchi T, Sobue T, Kitamura T (2019). Smoking, alcohol consumption, and risks for biliary tract cancer and intrahepatic bile duct cancer. J Epidemiol.

[8] McGee EE, Jackson SS, Petrick JL (2019). Smoking, alcohol, and biliary tract cancer risk: a pooling project of 26 prospective studies. J Natl Cancer Inst.

[9] Jusakul A, Cutcutache I, Yong CH (2017). Whole-genome and epigenomic landscapes of etiologically distinct subtypes of cholangiocarcinoma. Cancer Discov.

[10] Nakamura H, Arai Y, Totoki Y (2015). Genomic spectra of biliary tract cancer. Nat Genet.

[11] Yagyu K, Kikuchi S, Obata Y (2008). Cigarette smoking, alcohol drinking and the risk of gallbladder cancer death: a prospective cohort study in Japan. Int J Cancer.

[12] Tamakoshi A, Ozasa K, Fujino Y (2013). Cohort profile of the japan collaborative cohort study at final follow-up. J Epidemiol.

[13] Ikeda A, Miyashiro I, Nakayama T, Ioka A, Tabuchi T, Ito Y, Tsukuma H (2013). Descriptive epidemiology of bile duct carcinoma in Osaka. Jpn J Clin Oncol.

[14] Ye XH, Huai JP, Ding J, Chen YP, Sun XC (2013). Smoking, alcohol consumption, and the risk of extrahepatic cholangiocarcinoma: a meta-analysis. World J Gastroenterol.

[15] Akiba S (1994). Analysis of cancer risk related to longitudinal information on smoking habits. Environ Health Perspect.

[16] Wistuba II, Gazdar AF (2004). Gallbladder cancer: lessons from a rare tumour. Nat Rev Cancer.

[17] Lugo A, Peveri G, Gallus S (2020). Should we consider gallbladder cancer a new smoking-related cancer? A comprehensive meta-analysis focused on dose-response relationships. Int J Cancer.

[18] Hecht SS, Hatsukami DK (2022). Smokeless tobacco and cigarette smoking: chemical mechanisms and cancer prevention. Nat Rev Cancer.

[19] Khan SA, Carmichael PL, Taylor-Robinson SD, Habib N, Thomas HC (2003). DNA adducts, detected by 32P postlabelling, in human cholangiocarcinoma. Gut.

[20] Osaki T, Lin Y, Sasahira N, Ueno M (2022). Prevalence estimates of Helicobacter species infection in pancreatic and biliary tract cancers. Helicobacter.

